# Supplementary material for: Older adults and stroke survivors are steadier when gazing down
Source: PLoS One. 2023 May 19;18(5):e0285361. doi: 10.1371/journal.pone.0285361 (PMC10198484; doi:10.1371/journal.pone.0285361)
Supplement: S1 Table. Main results — (DOCX) [file pone.0285361.s002.docx]

**Table 2.** Results for all models

| Parameter | Main effect of Group | | Main effect of Condition | | Interaction | | R^2^ |
| --- | --- | --- | --- | --- | --- | --- | --- |
| Drs | F_2,860_=6.4 | P=0.002 | F_4,860_=163.8 | P<0.001 | F_8,860_=1.98 | P=0.046 | 0.34 |
| Dys | F_2,860_=6.6 | P=0.001 | F_4,860_=135.5 | P<0.001 | F_8,860_=1.2 | P=0.32 | 0.33 |
| Dxs | F_2,860_=4.3 | P=0.014 | F_4,860_=127.2 | P<0.001 | F_8,860_=2.4 | P=0.014 | 0.28 |
| Power | F_2,860_=8.1 | P<0.001 | F_4,860_=6.9 | P<0.001 | F_8,860_=1.1 | P=0.33 | 0.12 |

*The effect size R^2^ represents the marginal pseudo R^2^, as described by Nakagawa & Schielzeth [1], which represents the variance explained by the Fixed effects in the model.

[1] Nakagawa S, Schielzeth H. A general and simple method for obtaining R2 from generalized linear mixed-effects models. Methods Ecol Evol 2013;4(2):133-142.

stylefix
